# Genomic Exploration of Distinct Molecular Phenotypes Steering Temozolomide Resistance Development in Patient-Derived Glioblastoma Cells

**DOI:** 10.3390/ijms242115678

**Published:** 2023-10-27

**Authors:** Federica Fabro, Trisha V. Kers, Kate J. Feller, Cecile Beerens, Ioannis Ntafoulis, Ahmed Idbaih, Maite Verreault, Kate Connor, Archita Biswas, Manuela Salvucci, Jochen H. M. Prehn, Annette T. Byrne, Alice C. O’Farrell, Diether Lambrechts, Gonca Dilcan, Francesca Lodi, Ingrid Arijs, Andreas Kremer, Romain Tching Chi Yen, Miao-Ping Chien, Martine L. M. Lamfers, Sieger Leenstra

**Affiliations:** 1 Department of Neurosurgery Rotterdam, Brain Tumor Center, Erasmus Medical Center Cancer Institute, Erasmus Medical Center, Wytemaweg 80, Ee2236, 3015 CN Rotterdam, The Netherlands; f.fabro@erasmusmc.nl (F.F.); t.kers@erasmusmc.nl (T.V.K.); i.ntafoulis@erasmusmc.nl (I.N.); m.lamfers@erasmusmc.nl (M.L.M.L.); 2 Department of Molecular Genetics, Erasmus University Medical Center, 3015 CN Rotterdam, The Netherlands; k.feller@erasmusmc.nl (K.J.F.); c.beerens@erasmusmc.nl (C.B.); m.p.chien@erasmusmc.nl (M.-P.C.); 3 DMU Neurosciences, Service de Neurologie 2-Mazarin, Sorbonne Université, AP-HP, Institut du Cerveau—Paris Brain Institute—ICM, CNRS, University Hospital La Pitié Salpêtrière—Charles Foix, Inserm, F-75013 Paris, France; ahmed.idbaih@gmail.com (A.I.); maite.verreault@icm-institute.org (M.V.); 4 Department of Physiology and Medical Physics, Centre for Systems Medicine, Royal College of Surgeons in Ireland, D02 YN77 Dublin, Ireland; kateconnor@rcsi.ie (K.C.); architabiswas@rcsi.ie (A.B.); manuelasalvucci@rcsi.ie (M.S.); jprehn@rcsi.ie (J.H.M.P.); annettebyrne@rcsi.ie (A.T.B.); aliceofarrell@rcsi.ie (A.C.O.); 5 Department of Human Genetics, Laboratory for Translational Genetics, VIB Center for Cancer Biology, Katholieke Universiteit Leuven, 3000 Leuven, Belgium; diether.lambrechts@kuleuven.be (D.L.); gonca.dilcandurdag@kuleuven.be (G.D.); francesca.lodi@kuleuven.be (F.L.); ingrid.arijs@kuleuven.be (I.A.); 6 Information Technologies for Translational Medicine, L-4354 Esch-Sur-Alzette, Luxembourg; andreas.kremer@ittm-solutions.com (A.K.); romain.tching@ittm-solutions.com (R.T.C.Y.); 7 Luxembourg Centre for Systems Biomedicine, University of Luxembourg, L-4362 Esch-Belval Esch-Sur-Alzette, Luxembourg; 8 Oncode Institute, 3521 AL Utrecht, The Netherlands

**Keywords:** glioblastoma, temozolomide resistance, tumor heterogeneity, single-cell RNA sequencing

## Abstract

Chemotherapy using temozolomide is the standard treatment for patients with glioblastoma. Despite treatment, prognosis is still poor largely due to the emergence of temozolomide resistance. This resistance is closely linked to the widely recognized inter- and intra-tumoral heterogeneity in glioblastoma, although the underlying mechanisms are not yet fully understood. To induce temozolomide resistance, we subjected 21 patient-derived glioblastoma cell cultures to Temozolomide treatment for a period of up to 90 days. Prior to treatment, the cells’ molecular characteristics were analyzed using bulk RNA sequencing. Additionally, we performed single-cell RNA sequencing on four of the cell cultures to track the evolution of temozolomide resistance. The induced temozolomide resistance was associated with two distinct phenotypic behaviors, classified as “adaptive” (ADA) or “non-adaptive” (N-ADA) to temozolomide. The ADA phenotype displayed neurodevelopmental and metabolic gene signatures, whereas the N-ADA phenotype expressed genes related to cell cycle regulation, DNA repair, and protein synthesis. Single-cell RNA sequencing revealed that in ADA cell cultures, one or more subpopulations emerged as dominant in the resistant samples, whereas N-ADA cell cultures remained relatively stable. The adaptability and heterogeneity of glioblastoma cells play pivotal roles in temozolomide treatment and contribute to the tumor’s ability to survive. Depending on the tumor’s adaptability potential, subpopulations with acquired resistance mechanisms may arise.

## 1. Introduction

The emergence of therapy resistance in glioblastoma still undermines the chance of patients’ survival. Unfortunately, after standard therapy that includes surgery, radiotherapy, and chemotherapy with temozolomide (TMZ), relapses inevitably occur [1,2]. This is mainly caused by the emergence of therapy resistance, which diminishes the cytotoxic effects of chemotherapy. Temozolomide resistance is a multifaceted challenge with several underlying mechanisms, the foremost being the activity of the O6-methylguanine-DNA methyltransferase (MGMT) protein, responsible for the removal of O6-methylguanine lesions induced by temozolomide [3]. Glioblastoma cells, however, have displayed remarkable adaptability in circumventing this toxicity through a diverse panel of mechanisms. These include the activation of alternative DNA repair systems, aberrant signaling pathways, the induction of autophagy, epigenetic modifications, microRNA regulation, and even the exchange of information through extracellular vesicles [4]. Increasing the understanding of the driving molecular characteristics involved in temozolomide resistance has become of utmost importance to improve treatment strategy and patient survival.

Drug resistance is a complex phenomenon that comprises intrinsic and acquired components [5]. Over the past years, many studies have tried to undercover the processes supporting temozolomide resistance in glioblastoma [4,6]. However, most of the in vitro studies investigating temozolomide resistance carry some limitations. One means to investigate therapy resistance includes the analysis of the paired primary and recurrent samples. However, this approach is often limited by the availability of the matched recurrent samples, as the benefit of repeated resections upon recurrence is still controversial [7]. Therefore, many studies rely on the administration of temozolomide to treatment-naïve cell cultures. The suitability of using in vitro cell culture as a model for further investigations is supported by the correlation observed between in vitro sensitivity to temozolomide and clinical outcome [8]. However, the exploration of resistance development based on tumor-specific drug concentrations has not yet been comprehensively investigated. In fact, another important source of complexity in glioblastoma derives from its heterogeneous nature, present both among and within tumors [9]. In the recent years, with the advent of the single-cell sequencing, the intra-tumor heterogeneity of glioblastoma has been more deeply characterized, including the identification of specific cellular states that recapitulate four distinct neural cell types [10]. Since then, several studies have been carried out at the single-cell level to reveal different aspects of glioblastoma heterogeneity, but only a few have investigated response to drugs [11,12,13,14,15,16,17]. Little is still known regarding glioblastoma heterogeneity in relation to temozolomide resistance and its development over time. Here, to recapitulate the intrinsic and acquired development of temozolomide resistance, we established a panel of 21 temozolomide-resistant patient-derived glioblastoma cell cultures through the prolonged low-dose exposure to temozolomide. Given the evidence of the crucial role of heterogeneity in glioblastoma, we explored the inter- and intra-tumoral diversity of temozolomide resistance, to investigate and provide a preliminary insight on the complex phenomenon of resistance and its development.

## 2. Results

### 2.1. Glioblastoma Cell Cultures Display Two Distinct Behavioral Phenotypes

**Figure 1:**
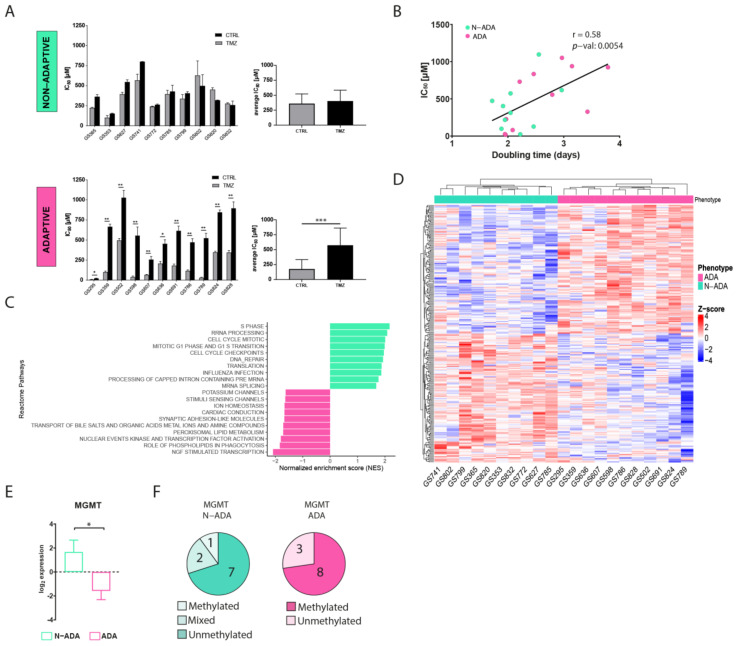
Development of temozolomide resistance in glioblastoma cell cultures. (**A**) IC50 values of untreated and temozolomide-treated N-ADA (top) and ADA (bottom) cell cultures. On the right, the average of the IC50 concentrations. (**B**) Dotplot displaying the correlation between the IC50 and doubling times of N-ADA and ADA cell cultures. (**C**) Barplot displaying the top 10 most significant enriched pathways containing the largest number of genes in N-ADA and ADA cell cultures. (**D**) Heatmap of the top 1% most correlated genes for the N-ADA and ADA cell cultures. (**E**) Boxplot showing the gene expression levels of MGMT. The + reports the mean. (**F**) Pie charts showing the amount of N-ADA and ADA cell cultures showing the methylated and unmethylated MGMT promoter status. * *p* < 0.05, ** *p* < 0.01, *** *p* < 0.001. CTRL: untreated; TMZ: temozolomide. MGMT: O6-methylguanine-DNA methyltransferase.

A total of 21 patient-derived primary wildtype glioblastoma cell cultures were cultured in medium containing ¼ of temozolomide half-maximal inhibitory concentration (IC50) for 90 days to induce resistance. The variation of IC50 was used as a parameter to assess the acquisition of resistance. After the treatment, the cell cultures displayed two distinct phenotypes. A total of 48% of the cell cultures were characterized by a similar IC50 (ranging from 102 to 566 µM in the untreated cells, and from 152 to 799 µM in the TMZ-treated cells) when compared to the untreated control, henceforth named non-adaptive (N-ADA) (Figure 1A). On the contrary, the remaining 52% of the cell cultures showed a significant increment (twofold or higher, ranging from 7.5 to 495 µM in the untreated cells, and from 23 to 1028 µM in TMZ-treated cells) of the IC50 after temozolomide treatment, henceforth named adaptive (ADA) (Figure 1A). In addition, the untreated N-ADA cells showed on average a twofold higher basal IC50 (average: 361 µM) than the untreated ADA cells (average: 176 µM), suggesting a prevalence of intrinsic resistant populations in the former. As expected from prolonged cell culturing, the IC50 values of untreated controls displayed variations over time. Nevertheless, 19/21 cell cultures could consistently be assigned to the same phenotypical category (Table S1).

To characterize the aggressiveness of the cell cultures prior to treatment and its possible relation with the two phenotypes, the growth rate was measured. A moderate positive correlation was observed between doubling time and temozolomide sensitivity of the cells prior to treatment (Figure 1B), indicating a tendency of the less proliferative cells to be more resistant to temozolomide.

### 2.2. ADA and N-ADA Glioblastoma Cells Express Distinct Molecular Phenotypes Prior to Treatment

To identify pathways and gene expression signatures underlying the ADA and N-ADA phenotypes, we analyzed the transcriptomic data of the cell cultures prior to treatment by employing gene set enrichment analysis (GSEA). The significantly enriched pathways in the N-ADA group were linked to cell cycle regulation, DNA repair, mRNA and rRNA processing, and translation (Figure 1C). These features have been found to be closely related to aggressiveness and intrinsic drug resistance in glioblastoma [18,19]. On the other hand, the ADA group was enriched for pathways associated with the transcriptional regulation of neuronal system development, lipid-associated pathways, and transport of small molecules. Both neurodevelopment and transcription regulators are known to play a key role in tumorigenesis and the plasticity of glioma stem cells (GSC), which are considered the main drivers of glioblastoma heterogeneity [20]. Metabolic reprogramming is recognized as a hallmark of cancer and a mechanism involved in the adaptation to various stimuli and conditions, requiring among the processes also the regulation of molecular trafficking [21,22]. Consistently, the top 1% most correlated genes of the N-ADA cell cultures contained genes related to cell cycle regulation and DNA repair (E2F2, FAAP24, CCND1, POLE2, TERT, and MGMT), while ADA cell cultures presented genes linked to development (EPAS1, F3, EGR3, SHH, NDN, HAP1) and metabolism including glucose and lipid metabolic genes (DGKG, GPD1, LCAT, ABCD2, ABCA8) (Figure 1D, Table S2). Among these genes, MGMT is known to play a crucial role in temozolomide resistance and was significantly upregulated in N-ADA cells (median log2 N-ADA: 3.1; median log2ADA: −2.6; *p*-value = 0.0157) (Figure 1E) [6]. Coherently, 70% of the N-ADA and 72% of the ADA cell cultures grouped with the unmethylated and methylated status, respectively, of the MGMT promoter (Figure 1F).

Thus, the development of temozolomide resistance resulted in two distinct phenotypical behaviors prior to treatment, suggesting the prevalence of a primary resistance in the N-ADA cells, and an adaptation potential in the ADA cells.

### 2.3. ADA Cell Cultures Are Characterized by Subpopulations Taking Over during Temozolomide Resistance

In order to examine the temporal changes and heterogeneity of temozolomide resistance within cell cultures displaying increased resistance, we conducted single-cell RNA sequencing at three key stages: prior to treatment (T0), after the first exposure to temozolomide (T1), and following 90 days of temozolomide treatment (T2). This analysis focused on two ADA cell cultures, GS359 and GS789. These cell cultures showed the highest IC50 increase over temozolomide treatment following the 90 days of drug treatment. For comparison purposes, we included two N-ADA cell cultures: one encompassing all three time points (GS785) and another covering the first two time points (GS772).

**Figure 2:**
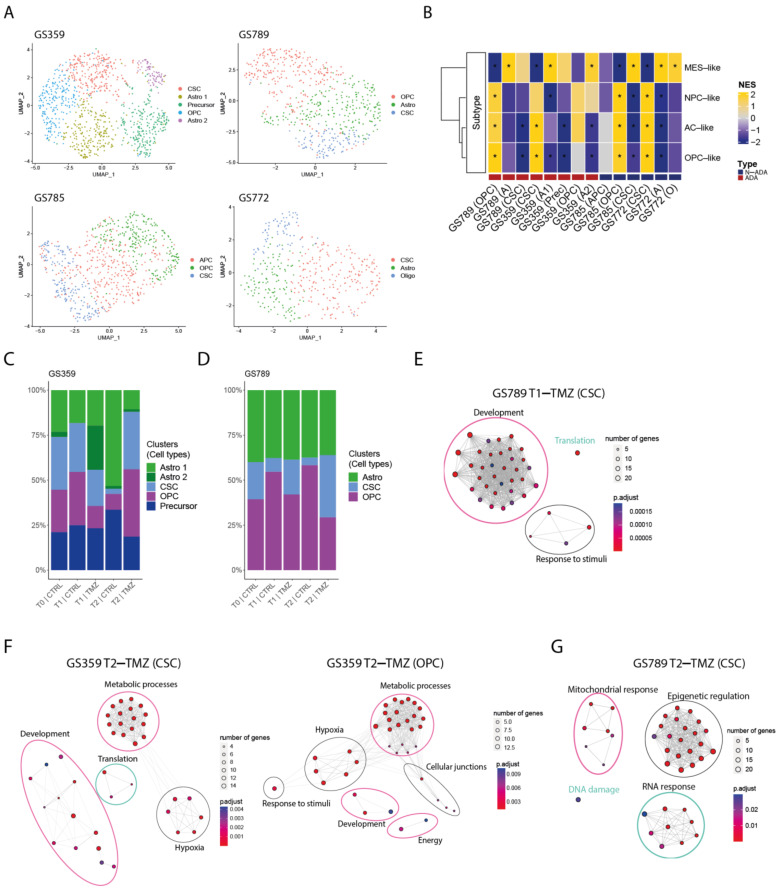
Intra- and inter-tumoral heterogeneity in ADA cell cultures. (**A**) UMAP plot of GS359, GS789, GS785, and GS772 annotated by glioma-related cell types. (**B**) Heatmap of Neftel cellular states subtypes across all clusters and samples. Red and blue bottom legends refer to ADA and N-ADA derivation, respectively. * indicates *p* < 0.05 (**C**) Bar plot displaying the composition of clusters and their relative cell-type annotation for GS359. (**D**) Bar plot displaying the composition of clusters and their relative cell-type annotation for GS789. (**E**) Network graphs displaying the enriched biological processes identified in T1-TMZ CSC cluster of GS789. (**F**) Network graphs displaying the enriched biological processes identified in T2-TMZ CSC (left) and OPC (right) clusters of GS359. (**G**) Network graphs displaying the enriched biological processes identified in T2-TMZ CSC cluster of GS789.| The thickness of the lines in the network graphs represents the percentage of the overlapping genes. Ovals identify the category of the major biological processes related to the clustered processes. Specifically, pink ovals describes ADA-related mechanisms, green ovals represents N-ADA-related mechanisms, and black ovals represents neither ADA nor N-ADA-related mechanisms. CTRL: untreated; TMZ: temozolomide; OPC: oligodendrocyte progenitor-like; Astro/A: astrocytes-like; Oligo: oligodendrocyte-like; CSC: cancer stem cells. MES: mesenchymal-like; NPC: neural progenitor-like; AC: astrocyte-like; NES: Normalized Enrichment Score; *: significant, O: Oligo-like; Prec: Precursors.

To characterize the composition of the cell cultures, we identified and characterized the main subpopulations present within each sample. Multiple subpopulations were identified and annotated based on glioblastoma-related cell-type markers (Figure 2A). In addition, we examined the enrichment for Neftel cellular states. Both ADA and N-ADA cell cultures comprised subpopulations exhibiting a mixture of cellular states (Figure 2B). The copy number variation (CNV) profile of the tumor cells remained mostly consistent throughout the cell culturing period, as evidenced by the retained CNV profile (Figure S1).

During the temozolomide exposure of ADA cell cultures, we observed a progressive increase in one or more subpopulations over time. In GS359, the CSC and OPC subpopulations showed a marked increase in temozolomide-treated cells (CSC: 32%; OPC: 37.3%) compared to untreated controls (CSC: 2.6%; OPC: 7.9%) (Figure 2C). At the point of resistance, the CSC and OPC subpopulations expanded 12 and 4.7 times more, respectively, compared to untreated cells. Similarly, in GS789, the CSC subpopulation showed continuous expansion in temozolomide-treated cells (34.4%) compared to the untreated condition (4.1%) (Figure 2D).

Conversely, N-ADA cell cultures exhibited a different behavior. In GS785, although the Astro subpopulation was the most abundant, no difference was observed compared to the untreated condition (Figure S2A). The only subpopulation that increased in temozolomide-treated samples was the OPC, with a 2.2-fold increase compared to untreated cells (untreated: 8.7%; treated: 19.4%). In GS772, the subpopulation composition remained unchanged at the beginning of treatment, with the CSC subpopulation consistently being the most abundant (45.2%) (Figure S2C).

In summary, ADA cell cultures demonstrated an adaptive behavior, with subpopulations increasing in response to temozolomide treatment, constituting a significant proportion of the tumor cell population. Conversely, N-ADA cell cultures displayed a non-adaptive characteristic, exhibiting less-pronounced changes in subpopulation composition during treatment.

### 2.4. Dominant Temozolomide-Resistant Subpopulations of ADA Cell Cultures Are Characterized by Distinct Resistant Biological Processes

In order to gain insight into the behavior of the dominant subpopulations in temozolomide-resistant cell cultures during treatment (CSC and OPC in GS359, CSC in GS789), we conducted an analysis of differentially expressed genes over time (Table S3) and their association with biological processes. Using the significantly upregulated genes identified in the dominant temozolomide-resistant subpopulations, we performed gene enrichment analysis to uncover the inter- and intra-tumoral heterogeneity of resistance mechanisms.

At the initial exposure to temozolomide (T1), the CSC subpopulation of GS359 exhibited upregulation of three genes (CHAC1, ARL6IP1, and MORN4), while the OPC subpopulation did not show significant upregulation. In GS789, the CSC subpopulation displayed upregulation of genes associated with developmental processes, translation, and response to stimuli (Figure 2E).

**Figure 3:**
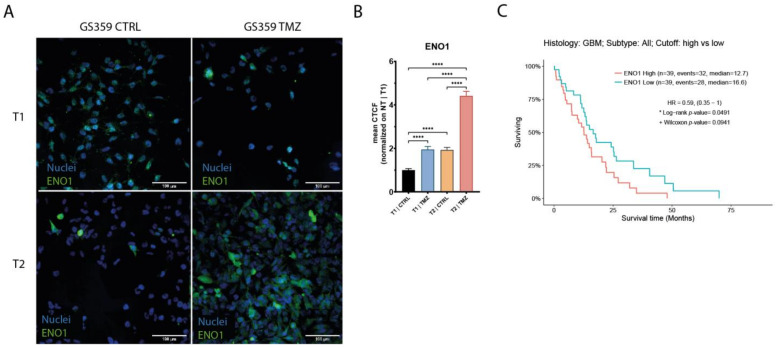
Expression of ENO1 in GS359 ADA cell culture. (**A**) Representative panel of the immunofluorescence staining of ENO1 in G359 at different conditions (T1-CTRL, T1-TMZ, T2-CTRL, T2-TMZ). (**B**) Bar plot represents the normalized amount of ENO1 protein expression during temozolomide treatment. **** *p* < 0.0001. (**C**) Kaplan–Meier graphs showing the overall survival related to high and low expression of ENO1 derived from the TCGA-GBM RNA-seq dataset. T1-TMZ: first temozolomide exposure; T1-CTRL: untreated cells at first temozolomide exposure; T2-TMZ: temozolomide resistant; T2-CTRL: untreated cells at temozolomide resistance. CTCF: corrected total cell fluorescence.

Upon the establishment of temozolomide resistance (T2), the subpopulations exhibited more distinct signatures. Both the CSC and OPC subpopulations of GS359 shared genes involved in development, metabolic processes, and hypoxia (Figure 2F and Figure S3A,B). Additionally, subpopulation-specific processes were identified, including translation in the CSC subpopulation, and the response to stimuli, cell junctions, and cell energy in the OPC subpopulation (Figure S3A,B). Among the common genes in both subpopulations, ENO1 was identified, a gene associated with metabolic and hypoxia processes known to promote cell growth, migration, and invasion in glioblastoma [23]. Protein-level analysis revealed increased ENO1 expression over time (fold change: 4.4; *p*-value < 0.0001) (Figure 3A,B). Moreover, high expression of ENO1 was correlated with lower overall survival in glioblastomas (Figure 3C).

In the CSC subpopulation of GS789, upregulation of genes involved in mitochondrial processes, RNA processes, DNA damage response, and epigenetic regulation, including methylation, was observed (Figure 2E and Figure S3C).

We further conducted an analysis of ADA cell culture development over time by reconstructing pseudotime trajectories. Our findings revealed unique trajectories specific to each cell culture (Figure S4A,B). Notably, in GS359, the two prevalent TMZ-related subpopulations remained closely aligned with their initial states, suggesting a dynamic mixture of cellular fates. Conversely, in GS789, the prominent TMZ-resistant subpopulation exhibited the furthest deviation from its initial state, indicating a more pronounced transformation in the majority of cells after TMZ treatment.

Although the N-ADA cell culture GS785 displayed fewer changes in subpopulation composition, temozolomide-specific responses, particularly mitochondrial processes, were still evident in all subpopulations (Figure S2B). In GS772, fewer upregulated genes were identified after the initial exposure to temozolomide, indicating only a minor initial influence of temozolomide on the tumor cells (Figure S2D).

In summary, the results from the dominant subpopulations in ADA cell cultures revealed heterogeneous pathway development involving common genes and biological processes. Despite exhibiting specific responses to temozolomide, both ADA and N-ADA phenotypes appeared to contribute to the development of temozolomide resistance in ADA cell cultures.

### 2.5. Markers of Temozolomide Resistance Are Found in Recurrent Glioblastoma Tumors

**Figure 4:**
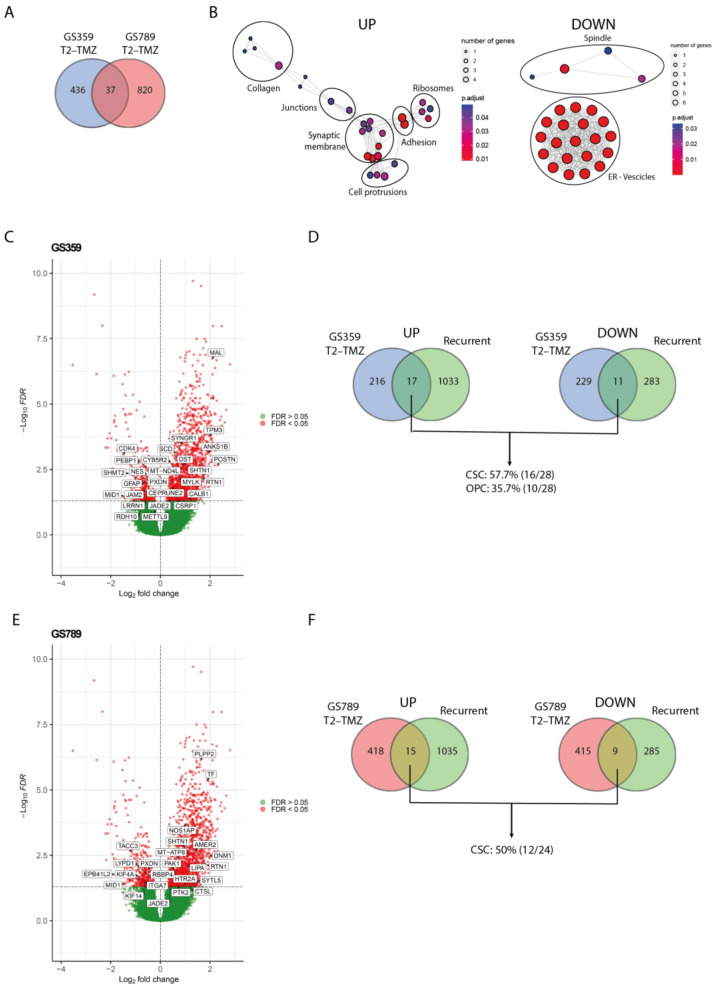
Validation of ADA resistance signature with recurrent glioblastoma. (**A**) Venn diagram displaying the amount of overlapping temozolomide-resistant gene signature (T2-TMZ) of GS359 and GS789. (**B**) Network graphs displaying the enriched cellular compartments identified in the common 37 genes of temozolomide resistant (T2-TMZ) ADA cell cultures. On the left, the network graph is related to the upregulated genes, while on the right the network graph refers to the downregulated genes. (**C**) Volcano plot representing the differentially expressed genes between primary and recurrent glioblastomas derived from the GLASS dataset. Positive log2 fold changes refer to the upregulation in the recurrent tumors compared to the primary glioblastomas. The overlapping significant up- and downregulated genes with temozolomide-resistant (T2-TMZ) GS359 are displayed in the white boxes. (**D**) Venn diagrams displaying the amount of overlapping up- (left) and downregulated (right) genes between recurrent and temozolomide-resistant (T2-TMZ) GS359, and the percentage of the genes related to the two dominant clusters. (**E**) Volcano plot representing the differentially expressed genes between primary and recurrent glioblastomas derived from the GLASS dataset. The overlapping significant up- and downregulated genes with temozolomide-resistant (T2-TMZ) GS789 are displayed in the white boxes. (**F**) Venn diagrams displaying the amount of overlapping up- (left) and downregulated (right) genes between recurrent and temozolomide-resistant (T2-TMZ) GS789, and the percentage of the genes related to the dominant cluster. The thickness of the lines in the network graphs represents the percentage of the overlapping genes. Black ovals identify the major cellular compartment category related to the clustered compartments. TMZ: temozolomide.

To validate the clinical relevance and identify a common pattern in the ADA phenotype, we conducted an in silico study using the GLASS database to investigate the expression of ADA temozolomide-resistant markers in recurrent glioblastoma samples. We considered all subpopulations (pseudo-bulk) to determine the overall markers of temozolomide resistance (Table S4). As shown in Figure 4A, we found that 37 genes (18 upregulated and 19 downregulated) were commonly shared between GS359 and GS789 (Table S5).

While these genes may not be directly implicated in a particular biological process, the upregulated genes exhibited significant enrichment in cellular components such as collagens, junction complexes, synaptic membranes, filopodia and lamellipodia protrusions, adhesions, and ribosomes (Figure 4B). This suggests their potential involvement in functions like migration, cell communication, and protein synthesis. Conversely, the downregulated genes were enriched for spindle, endoplasmic reticulum (ER) and vesicle compartments (Figure 4B).

By comparing the differential gene expression between primary and recurrent wildtype glioblastoma with the 37 genes commonly shared between the two ADA cultures, we identified 3 overlapping upregulated genes (SHTN1, RTN1, JADE2) and 2 downregulated genes (PXDN, MID1). SHTN1 is known to regulate neuronal migration and is associated with nervous system development [24]. RTN1 plays a role in neuroendocrine membrane trafficking, and JADE2 is involved in histone acetylation and chromatin organization [25,26]. PXDN is a peroxidase involved in the extracellular matrix formation, while MID1 is an E3 ubiquitin ligase implicated in neurodevelopment [27,28]. The limited overlap of genes suggests the presence of more tumor-specific patterns associated with resistance and recurrence. To explore this further, we investigated the overlapping differentially expressed genes between primary and recurrent glioblastoma in each cell culture separately (Table S5). In GS359, the amount of overlapping genes increased to 17 upregulated and 11 downregulated genes (Figure 4C), with 57.7% and 35.7% originating from the dominant temozolomide-resistant subpopulations CSC and OPC, respectively (Figure 4D). Similarly, in GS789, we found 15 upregulated and 9 downregulated genes shared with the recurrent samples (Figure 4E), 50% of which originated from the dominant CSC temozolomide-resistant subpopulation (Figure 4F).

We adopted the same approach to examine the T2-TMZ marker genes in N-ADA cell cultures GS785. In comparison to the ADA cell cultures, we observed a similar number of genes detected in recurrent glioblastoma (Figure S5A). Lacking a dominant temozolomide-specific subpopulation, we investigated all subpopulations. Interestingly, we observed fewer overlapping genes compared to the ADA-dominant subpopulations, with 36.4%, 18.2%, and 0% of the genes across all subpopulations (Figure S5B). Likewise, we noted a comparable pattern in GS772 at the initiation of treatment, where the impact of temozolomide is not prominently evident (Figure S5C).

Overall, the markers of temozolomide resistance of ADA cell cultures that were identified in recurrent glioblastoma specimens suggest to reflect the cells’ adaptive response to the drug, indicating the acquisition of new mechanisms to evade its toxicity. These markers exhibit tumor-specific characteristics, underscoring their relevance to individual glioblastomas, and are also enriched for markers associated with the predominant temozolomide-resistant subpopulations. The absence of a temozolomide-specific subpopulation in N-ADA cell cultures suggests, instead, a more cooperative response among all tumor cells, in contrast to ADA cell cultures where resistance primarily arise from cellular adaptation of specific subpopulations.

## 3. Discussion

Temozolomide resistance is a persistent issue in glioblastoma treatment. Chemotherapy resistance can be attributed to the heterogeneous nature of glioblastoma, characterized by the presence of multiple subpopulations that do not respond to, or escape therapy [29].

The first experimental evidence of this study indicated the presence of two temozolomide resistance behavioral phenotypes. The N-ADA phenotype displayed a phenotypical behavior close to pre-existing resistance phenotypes, where the regulation of cell cycle, DNA repair, RNA processing, and protein synthesis played a major role. Cells use an interconnected network of pathways that regulate cell cycle, DNA repair and replication, as well as transcriptional processes, known as the DNA-damage response (DDR), to preserve the integrity of the genome, leading to cell survival after genotoxic stresses [30]. These features, in addition to the detection of the unmethylated MGMT in most N-ADA samples, further supported the presence of a main intrinsic resistance signature. In contrast, the ADA cell cultures were mainly characterized by the presence of neurodevelopmental, metabolic, and transport signatures. The main drivers of developmental processes lie in the stem-like population, which has the ability and plasticity to drive tumor growth and heterogeneity [31]. In parallel, the integration of intracellular transport and metabolism becomes a central process of cellular adaptation to environmental changes and cancer progression [22]. Therefore, the characteristics of ADA cell cultures prior to treatment suggest a crucial role for the ability to adapt to external perturbations, such as temozolomide treatment.

With the emergence of single-cell sequencing techniques, it has become possible to delve deeper into tumor analysis, enabling molecular analysis of distinct subpopulations. The intra-tumoral heterogeneity observed in the cell cultures appeared to be partially dependent on an adaptive phenotype, as indicated by the increase in specific subpopulations over time in ADA tumors under temozolomide treatment, which was not observed in N-ADA cell cultures. This expansion of subpopulations aligns with the adaptability phenotype of the cell cultures. Interestingly, in both ADA cell cultures, the common expanded subpopulation was the cancer stem cell (CSC) population. Despite the cell culture-specific molecular characteristics, ADA-related mechanisms remained prevalent in ADA subpopulations. The ADA subpopulations were characterized by the upregulation of developmental and metabolic genes, which acted as triggers and mediators of resistance. It has also been recognized that developmental signals are responsible for maintaining the CSC population, as CSCs exhibit high metabolic plasticity and can cause therapy resistance through dynamic transitions between distinct metabolic phenotypes [32,33]. In glioblastoma, different cellular states have been identified along the neurodevelopmental and metabolic axes, which are associated with survival [34]. Our results additionally suggest that developmental and metabolic features are not only important in sustaining the adaptability and heterogeneity of glioblastoma cells but also in supporting the development of temozolomide resistance in certain subpopulations. Among the markers of resistance, we confirmed the upregulation of ENO1, which is not only a marker of glycolysis but is also involved in other pathological processes such as hypoxia. ENO1 serves as a relevant marker of glioblastoma aggressiveness and poor prognosis [23]. In the N-ADA cell culture, we did not observe the upregulation of N-ADA specific mechanisms; instead, we observed a variety of different mechanisms, suggesting that their intrinsic characteristics were not utilized and adapted to cope with the drug.

Among the identified subpopulations, we focused on the dominant ones that were most relevant to temozolomide resistance. It is not surprising that the escape mechanisms developed by the resistant subpopulations showed the greatest variability among the cell cultures, each displaying a unique pattern of molecular response to temozolomide. When investigating a common resistance signature among ADA-resistant cell cultures, we identified genes associated with neurodevelopment, particularly those involved in cell protrusions, communication, and extracellular matrix organization. Recent studies have revealed that the enrichment of cilium-related genes is characteristic of the glioblastoma of long-term survival patients [35]. In our study, we propose a new potential role for these genes in governing and regulating temozolomide response, along with cell communication signaling within the tumor environment. When comparing the presence of common temozolomide-resistant gene patterns with recurrent glioblastomas, we did not observe significant overlaps. However, when conducting a tumor-specific investigation, more matches were identified, indicating that the development of temozolomide resistance is likely highly tumor-specific. To validate our findings, in this study we employed the transcriptomic data of recurrent tumor samples. It is crucial to acknowledge that specimens obtained from recurrent patients are highly selected, representing a specific subset of cases as second surgeries are typically reserved for patients who have a more favorable prognosis. Moreover, it is important to consider that tumor tissues vary in cell-type populations, as they do not solely consist of tumor cells like our in vitro cell culture system. Additionally, recurrent glioblastomas have not only been exposed to temozolomide but also to radiation. Finally, the analysis was conducted on a subsample of tumor cells, and the limited number of tumors analyzed is not sufficient to draw definitive conclusions about the observed trends. Further experimental validations as well as validations in larger cohorts are, therefore, necessary. Nevertheless, despite these limitations, our study successfully identified significant genes relevant to temozolomide resistance in clinical samples.

Comprehensive tumor molecular characterization is crucial for identifying drug-resistance evolution patterns, as observed in the N-ADA and ADA phenotypes. Regular monitoring is essential to track phenotypic and molecular changes during treatment, aiding treatment decisions. Personalized therapeutic approaches targeting specific resistance pathways in combination with temozolomide may reduce relapse risk. These findings should stimulate further research on drug resistance and heterogeneity in glioblastoma, by including experimental investigations to elucidate population heterogeneity and behaviors while also considering microenvironmental factors, and identifying potential molecular targets. Such research holds promise for developing more effective therapies to prevent or overcome resistance in glioblastoma.

## 4. Materials and Methods

### 4.1. Cell Cultures

Glioma stem-like cell (GSC) cultures were obtained from our biobank containing cell cultures derived from primary brain tumor samples at the Erasmus Medical Centre as previously reported [36,37]. The use of patient material was approved by the local ethics committee of the hospital and all patients signed informed consent forms according to the guidelines of the Institutional Review Boards of Erasmus Medical Center.

The cultures were tested for mycoplasma infection using the MycoAlert PLUS Mycoplasma Detection Kit (Lonza, Rockland, ME, USA). The GSC cells were cultured using a validated neural stem culture medium proven to enrich for stem-like population [38,39]. Specifically, GSC cultures were grown in serum-free medium using Dulbecco’s modified Eagle’s medium-F12, supplemented with 1% Penicillin/Streptomycin, 2% B27, 20 ng/mL bFGF, 20 ng/mL EGF (all from Gibco, Thermo Fisher Scientific, Paisley, UK), and 5 μg/mL Heparin (Heysham, UK) in flasks coated with 1:100 diluted Cultrex PathClear Reduced Growth Factor BME (Cultrex, PathClear, Diblin, Ireland) [36].

### 4.2. Long-Term Temozolomide Treatment

Each cell culture was kept in medium containing ¼ IC50 concentration of temozolomide (Sigma Aldrich, Saint Louis, MO, USA) for 90 days. The medium with the drug was refreshed weekly. The treatment started with cell cultures ranging from passages 5 to 9 and extended up to passages 17 to 27.

### 4.3. Viability Assay

Temozolomide (Sigma Aldrich, Saint Louis, MO, USA) was dissolved in DMSO (Sigma Aldrich, Saint Louis, MO, USA). The cells were plated in a 96-well plate at a seeding density of 1000 cells/well in serum-free medium. The plates were incubated for 24 h prior to the drug treatment. After 24 h, serial 2- or 3-fold drug dilutions (0.0058–3 mM) were prepared in serum-free culture medium and added to each well in triplicates. The plates were incubated for an additional 5 days. Viability was measured with CellTiter Glo 2.0 (Promega, Madison, WI, USA), a luminescent ATP assay, according to manufacturer’s instructions. The luminescence was measured with the Infinite F Plex (Tecan, Giessen, The Netherlands). The percentage of viability was normalized based on the non-treated control.

### 4.4. Doubling Time

Cells were plated in a 96 well plate at a seeding density of 1000 cells/well, in triplicate, and incubated at 37 °C and 5% CO_2_. Cell counting was performed using a hemocytometer every 24 h for 9 days.

### 4.5. Methylation-Specific PCR for MGMT Promoter

All cell cultures were subjected to analysis of the MGMT promoter methylation status. In a separate study, the methylation status of the MGMT promoter in 13 samples was examined [8]. For the remaining 8 cell cultures, DNA extraction involved resuspending the cell pellets in 5% Chelex^®^100 (Bio-Rad, Lunteren, The Netherlands) and adding 10 mg/mL of proteinase K (Thermo Fisher Scientific, Carlsbad, CA, USA). The Chelex suspension was further heated for 8 min at 100 °C, vortexed, and centrifuged at 10,000× *g*. Following incubation and heating steps, the methylation-specific PCR was conducted using the supernatant obtained as previously mentioned.

### 4.6. Bulk RNA Sequencing and Data Analysis

Bulk RNA sequencing was performed on 21 primary glioblastoma cell cultures prior to treatment. The RNA library preparation and sequencing for all cell cultures was carried out as previously reported [40]. Briefly, a total of 2 μg of RNA was isolated from the cell cultures, using KAPA stranded mRNA-Seq kit (Kapa Biosystems, Nazareth, Belgium). The sequencing was single-end 50 bp performed on an Illumina HiSeq4000. Before mapping, the optical duplicates and adaptors were removed with Clumpify v.37.28 and Fastx clipper v.0.0.13, respectively. The quality of the reads was checked with FastQC v.0.11.4. Next, the reads were mapped to the human reference genome GrCh38 with STAR v.2.6 and gene expression matrices were generated with HTSeq v.0.10.0. The raw counts were normalized using the R package EdgeR [41]. The counts were filtered out for the lowly expressed genes and normalized using the TMM method in log2 scale. Batch effects were removed using the ComBat-seq package [42].

The enrichment pathway analysis was carried out with the Gene Set Enrichment Analysis (GSEA) v.4.1.0 software, using the Reactome database [43]. The correlation of the genes with the phenotype were ranked by their signal-to-noise ratio. Following the GSEA guidelines, FDR q-values < 0.25 were considered significant.

### 4.7. Single-Cell RNA Sequencing

For cell cultures GS359, GS785, and GS789, the cells sequenced comprised five conditions: prior treatment (T0), after the first TMZ exposure when cells reached the confluency (T1-TMZ and T1-CTRL) and after the last TMZ treatment (T2-TMZ and T2-CTRL). For cell culture GS772, the cell sequenced comprised three conditions: T0, T1-CTRL, and T1-TMZ. Cell cultures were washed with PBS and incubated with Accutase (Invitrogen, Carlsbad, CA, USA) until detachment, and collected in HBSS (Gibco, Paisley, UK). Subsequently, the cells were centrifuged and resuspended in HBSS and sorted (FACSAria II, BD Biosciences) into 384-well plates based on single-cell selection gating. Library preparation was carried out according to the CELseq2 protocol [44]. Cells were then subjected to single-cell transcriptomic sequencing (SORT-seq, ~100 k reads/cell). The sequencing and read alignment were performed as described by Muraro et al. [45]. In total, 4608 cells (each cell culture *n* = 1152) were sequenced.

### 4.8. Single-Cell RNA Sequencing Data Analysis

The downstream analysis of the scRNAseq data was performed in R using the Seurat package (v4.0.2) [46]. Each single-cell RNA sequencing dataset was independently analyzed, resulting in four distinct datasets. Initially, we filtered out low-quality cells. Specifically, cells containing 2500–10,000 features and less than 30% mitochondrial genes were selected. Subsequently, we applied data normalization using SCTransform, integrated all conditions including different time points and treatments, and performed cell cycle regression as implemented in Seurat. To identify cell clusters, we used Seurat’s FindClusters function on the integrated samples, and used UMAP for their visualization. Then, we identified differentially expressed genes of the clusters by applying the FindAllMarkers function with a non-parametric Wilcoxon rank-sum test (Bonferroni adjusted *p*-value < 0.05), aiming to pinpoint markers of drug response. The differential expression analysis was conducted separately for individual clusters as well as for merged (pseudobulk) clusters. The subpopulations were annotated using scCATCH package, using “Glioma” and “Glioblastoma” as type of human cancer tissue reference [47]. All the genes derived from the differential gene expression analysis identifying the clusters were used to create a ranked list that has been used as the input for the Gene Set Enrichment Analysis (GSEA) v.4.1.0 software [43]. GSEA was run using a module containing the signatures of the GBM cell states identified by Neftel et al. [10].

The differentially expressed genes identifying the drug treatments and time points response having an adjusted *p*-value < 0.05 were used to run the gene enrichment analysis for gene ontology (GO), biological processes (BP), or cellular components (CC). The network GO analysis was performed using the enrichGO and emapplot function of the clusterProfiler package [48].

The Gliovis webtool (http://gliovis.bioinfo.cnio.es/, accessed on 15 July 2021) was used to perform the survival analysis, choosing the TCGA-GBM RNA-seq dataset with “high vs. low” cut-off parameter [49].

The CNV were inferred using the package inferCNV, using the astrocytes derived from the study GSE171684 as normal cells [50,51].

The trajectory analysis and pseudotime calculation were performed in R using the package dyno, utilizing the Monocle DDRTree inference method [52].

### 4.9. Validation of Single-Cell RNA Sequencing

The markers of temozolomide resistance were validated using the GLASS database as a source of clinically relevant recurrent glioblastoma [53]. RNA sequencing transcript counts derived from the GASS database were downloaded from Synapse (https://www.synapse.org/glass, accessed on 5 May 2023) [53,54]. The samples were selected in order to retain the primary wildtype GBMs and their first recurrence. The preprocessing steps were carried out as described for the current dataset used in this study. Differential gene expression analysis was conducted using the R package EdgeR [41]. The significantly upregulated and downregulated genes were used for the overlapping with the marker genes of temozolomide resistance derived from the single-cell analyses.

### 4.10. Immunofluorescence Staining

Cells were grown on a glass coverslip until confluence. The immunofluorescence protocol was used for the detection and visualization of the protein of interest (ENO1). At each condition and time point, the cells were grown on glass coverslips. When they reached confluence, the cells were washed with PBS and fixed for 15 min with 4% PFA (Sigma Aldrich, The Netherlands). The cells were then rinsed with PBS three times, permeabilized in 0.3% Triton X-100 (Sigma Aldrich, The Netherlands), blocked in 5% goat serum (Abcam, Amsterdam, The Netherlands) for 1 h, and incubated with primary rabbit antibody (ENO1, Abcam, 1:200) for 2 h. The coverslips were then rinsed three times with 3% BSA in PBS. Next, the cells were incubated for 1 h with an Alexa Fluor 647 F(ab’)2-goat anti-rabbit IgG (H + L) antibody (Invitrogen, Carlsbad, CA, USA), diluted at 1:1000 as the secondary antibody. The coverslips were rinsed three times with 3% BSA in PBS and mounted on microscope slides using Vectashield hardset antifade mounting medium with DAPI (Vector Laboratories, Amsterdam, The Netherlands). Fluorescent images of immunolabeled cells were obtained using a CX7 confocal microscope (Thermo Fisher) with 20× objective, so that hundreds of cells per sample and per condition were available for analysis.

Images acquired with the CX7 confocal microscope (Thermo Fisher) were analyzed using the ImageJ software v1.53n. The DAPI signal and those from antibodies were used for segmentation of the nuclei and the cytoplasmic compartments, respectively, as well as the boundary of individual cells. After the segmentation, the area, the integrated density, and the mean gray value were measured. For each image, three background areas were used to correct against autofluorescence. For each cell, the corrected total cell fluorescence (CTCF) was calculated as described by McCloy et. al, with the following formula: CTCF = Integrated Density—(Area of selected cell X Mean fluorescence of background readings) [55]. The production on the bar graphs and statistical analysis (One-way ANOVA and Tukey’s multiple comparisons test) were performed using GraphPad Prism v.8.4.2.

### 4.11. Statistical Analysis

The half inhibitory concentration (IC50) was calculated using GraphPad Prism v.8.4.2 software, using a nonlinear regression analysis. The comparison between controls and treated IC50 were calculated in GraphPad applying paired Student’s *t*-test. The comparisons between the non-adaptive and adaptive group were calculated applying the unpaired *t*-test. A *p*-values of <0.05 was considered significant.

## Data Availability

Bulk RNA sequencing data of the cell lines (*n* = 21) applied in this article is available in the Gene Expression Omnibus Repository with Accession number GSE232173. Single-cell RNA sequencing data is available in the Gene Expression Omnibus Repository with Accession number: GSE226202.

## Supplementary Materials

The following supporting information can be downloaded at: https://www.mdpi.com/1422-0067/24/21/15678/s1.
