# Supplementary material for: Genomic Exploration of Distinct Molecular Phenotypes Steering Temozolomide Resistance Development in Patient-Derived Glioblastoma Cells
Source: Int J Mol Sci. 2023 Oct 27;24(21):15678. doi: 10.3390/ijms242115678 (PMC10647455; doi:10.3390/ijms242115678)
Supplement: Supplementary file 1 [file ijms-24-15678-s001.zip › Supplementary Figures_updated.pdf]

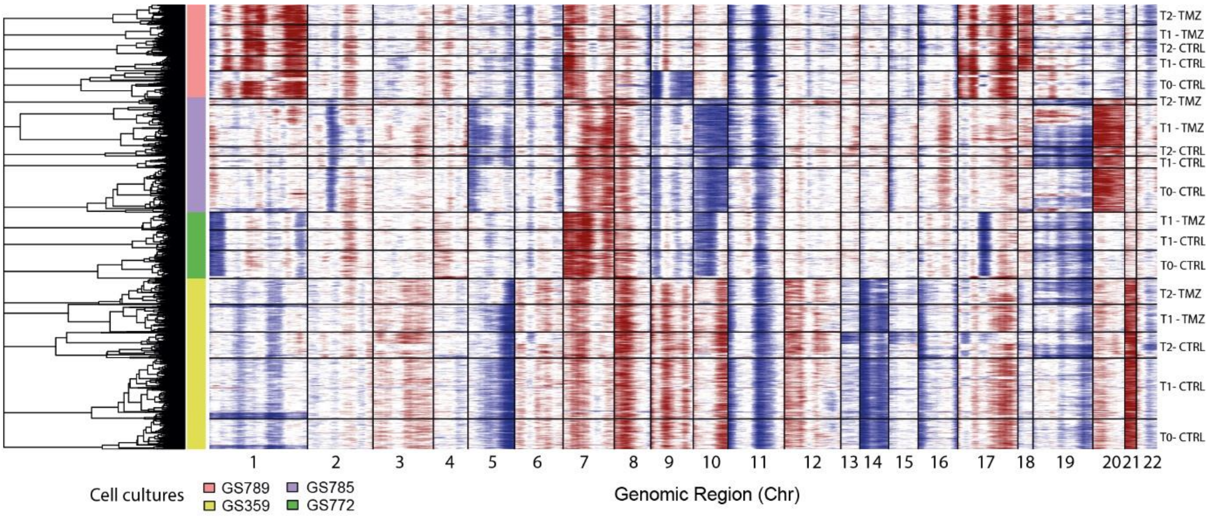


**Supplementary Figure S1. Copy number variation (CNV) profile of ADA and N-ADA cell cultures.** Inferred CNV profile of the cell cultures and conditions used in this study. T0-CTRL: initial untreated starting point. T1-TMZ: first temozolomide exposure; T1-CTRL: untreated cells at first temozolomide exposure; T2-TMZ: temozolomide resistant; T2-CTRL: untreated cells at temozolomide resistance..

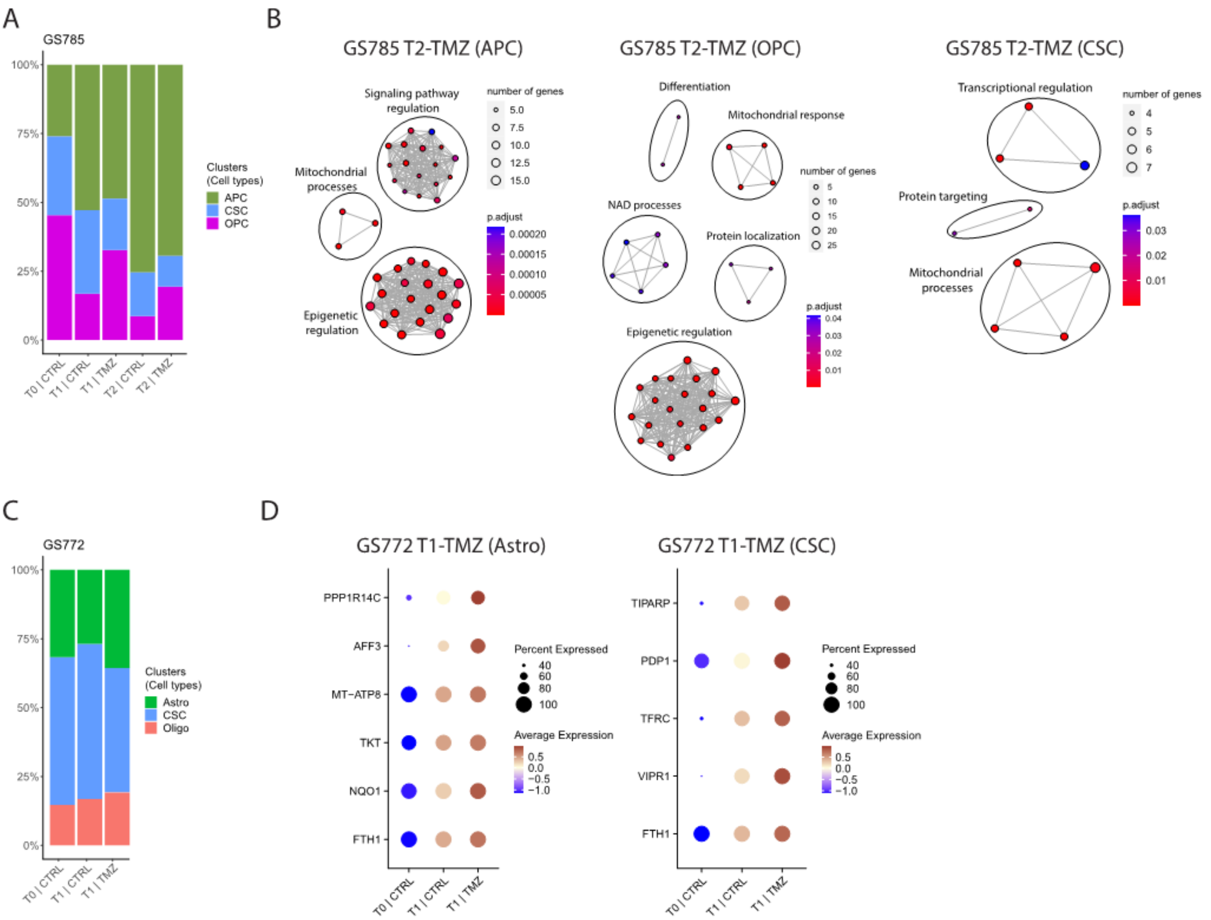


**Supplementary Figure S2. Intra- and inter-tumoral heterogeneity in N-ADA cell cultures.**

**(A)** Bar plot displaying the composition of clusters and their relative cell type annotation for GS785. **(B)** Network graphs displaying the enriched biological processes identified in T2-TMZ APC (left), OPC (middle) and CSC (right) clusters of GS785. **(C)** Bar plot displaying the composition of clusters and their relative cell type annotation

for GS772. **(D)** Dotplot displaying the upregulated genes identified in T1-TMZ Astro (left) and CSC (right) clusters of GS772. None were identified in the Oligo cluster. The thickness of the lines in the network graphs represents the percentage of the overlapping genes. Black ovals identify the major biological processes category related to the clustered processes. CTRL: untreated; TMZ: temozolomide; OPC: oligodendrocyte progenitor-like; APC: astrocyte progenitor-like; Astro: astrocytes-like; Oligo: oligodendrocyte-like; CSC: cancer stem cells.

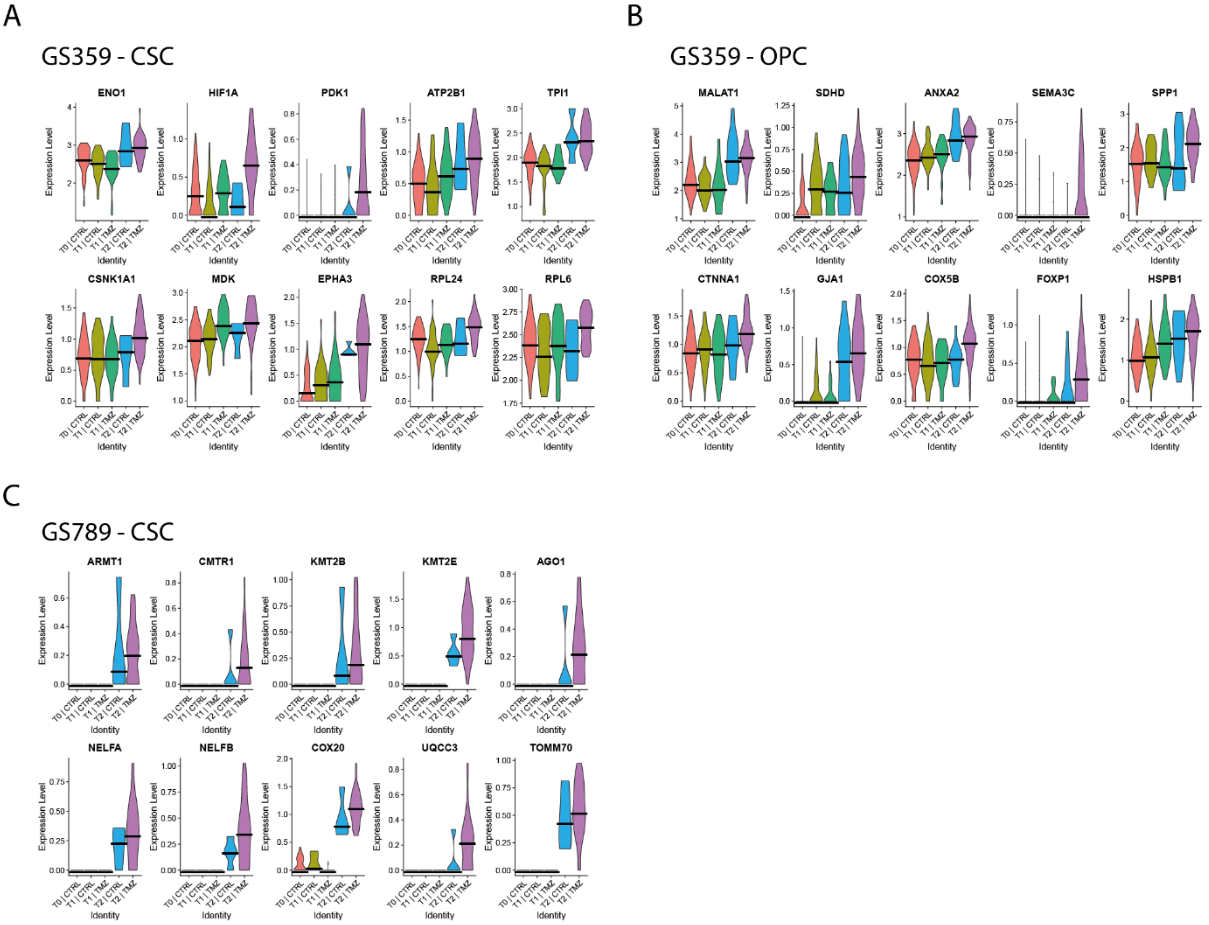


**Supplementary Figure S3. Expression of top 10 marker genes of the biological processes involved in the TMZ resistance in GS359 and GS789.**

**(A)** Violin plots of the gene markers upregulated in G359 CSC cluster at different conditions (T0-CTRL, T1-CTRL, T1-TMZ, T2-CTRL, T2-TMZ). The markers are involved in hypoxia (ENO1, HIF1A, PDK1, ATP2B1), metabolism (ENO1, TPI1, HIF1A), development (CSNK1A1, MDK, EPHA3) and translation (RPL24, RPL6). **(B)** Violin plots of the gene markers upregulated in G359 OPC cluster at different conditions (T0-CTRL, T1-CTRL, T1-TMZ, T2-CTRL, T2-TMZ). The markers are involved in hypoxia (MALAT1, SDHD), development (ANXA2, SEMA3C, SPP1), junctions (CTNNA1, GJA1), energy (COX6C, SDHD), and response to stimuli (FOXP1, HSPB1). **(C)** Violin plots of the gene markers upregulated in G789 CSC cluster at different conditions (T0-CTRL, T1-CTRL, T1-TMZ, T2-CTRL, T2-TMZ). The markers are involved in epigenetic regulation (ARMT1, CMTR1, KMT2B, KMT2E), RNA processing (AGO1, NELFA, NELFB), and mitochondrial regulation (COX20, UQCC3, TOMM70).

The black bars in the violins plot represent the median. T1-TMZ: first temozolomide exposure; T1-CTRL: untreated cells at first temozolomide exposure; T2-TMZ: temozolomide resistant; T2-CTRL: untreated cells at temozolomide resistance. CTCF: corrected total cell fluorescence.

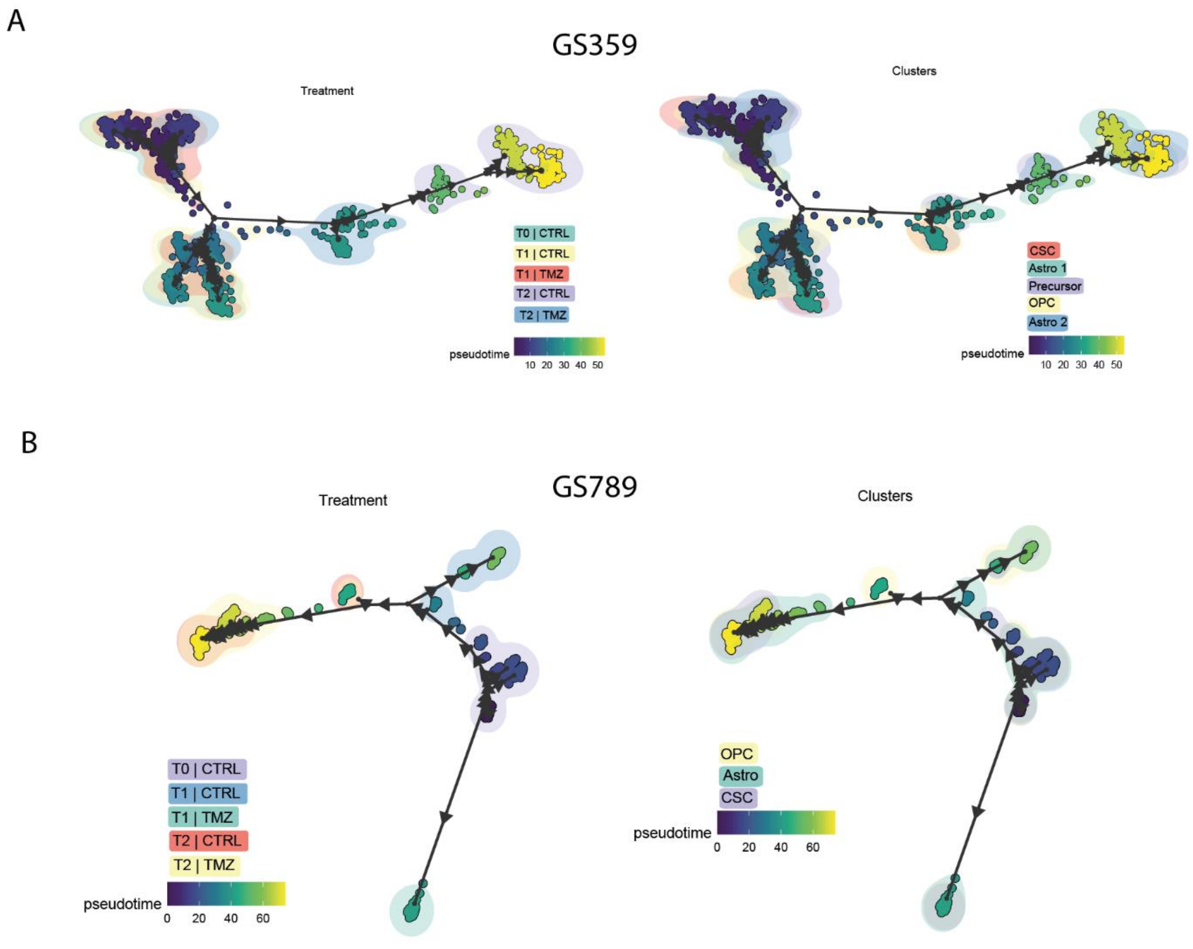


**Supplementary Figure S4**. Pseudotime trajectories of ADA cell cultures GS359 and GS789.

**(A)** Pseudotime trajectory of GS359 colored by condition (left) and by clusters (right). **(B)** Pseudotime trajectory of GS359 colored by clusters (left) and by clusters (right).
T1-TMZ: first temozolomide exposure; T1-CTRL: untreated cells at first temozolomide exposure; T2-TMZ: temozolomide resistant; T2-CTRL: untreated cells at temozolomide resistance. CTCF: corrected total cell fluorescence. CTRL: untreated; TMZ: temozolomide; OPC: oligodendrocyte progenitor-like; APC: astrocyte progenitor-like; Astro: astrocytes-like; Oligo: oligodendrocyte-like; CSC: cancer stem cells.

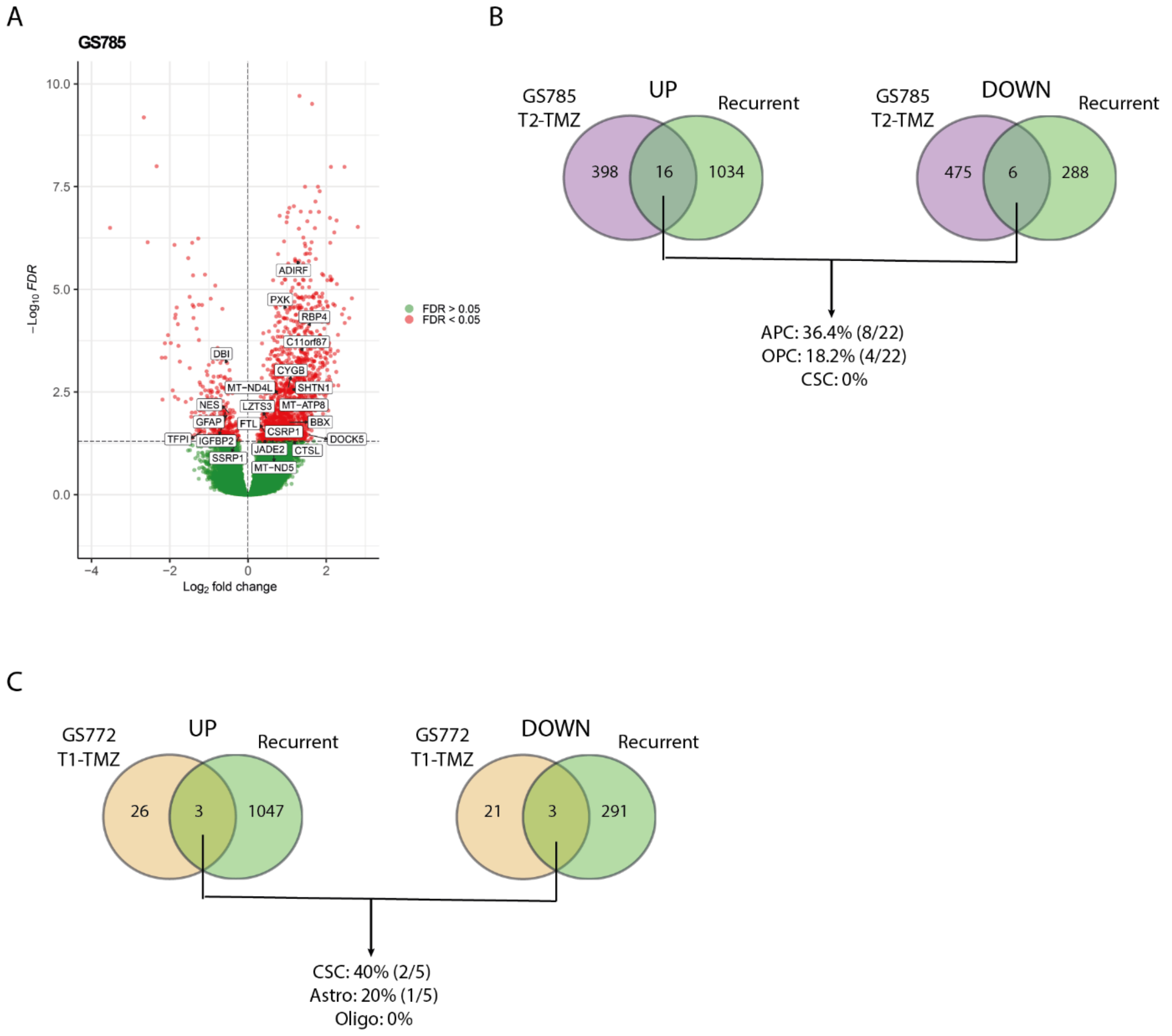


**Supplementary Figure S5**. **Validation of N-ADA resistance signature with recurrent GBMs.**

**(A)** Volcano plot representing the differentially expressed genes between primary and recurrent GBMs derived from the GLASS dataset. Positive log2 fold changes refer to the upregulation in the recurrent tumors compared to the primary GBMs. The overlapping significant up-and down- regulated genes with temozolomide resistant (T2-TMZ) GS785 are displayed in the white boxes. **(B)** Venn diagrams displaying the amount of overlapping up- (left) and down- (right) regulated genes between recurrent and temozolomide resistant (T2-TMZ) GS785, and the percentage of the genes related to the clusters composing GS785. **(C)** Venn diagrams displaying the amount of overlapping up- (left) and down- (right) regulated genes between recurrent and after the first exposure to temozolomide (T1-TMZ) GS772, and the percentage of the genes related to the clusters composing GS772.
TMZ: temozolomide; OPC: oligodendrocyte progenitor-like; APC: astrocyte progenitor-like; Astro: astrocytes-like; Oligo: oligodendrocyte-like; CSC: cancer stem cells.
